# Associations of perfluoroalkyl substances (PFAS) with lipid and lipoprotein profiles

**DOI:** 10.1038/s41370-023-00545-x

**Published:** 2023-04-05

**Authors:** Marianne Haug, Linda Dunder, P. Monica Lind, Lars Lind, Samira Salihovic

**Affiliations:** 1 School of Medical Sciences, Faculty of Medicine and Health, Örebro University, Örebro, Sweden; 2 Department of Medical Sciences, Cardiovascular Epidemiology, Uppsala Univeristy, Uppsala, Sweden; 3 Department of Medical Sciences, Occupational and Environmental Medicine, Uppsala University, Uppsala, Sweden

**Keywords:** Perfluoroalkyl substances; Human health; Lipoproteins; Cholesterol; Apolipoproteins; Protonnuclear magnetic resonance

## Abstract

**Background:**

Perfluoroalkyl substances (PFAS) are man-made chemicals with unique properties that are widely distributed in humans and the environment. Recent studies suggest that PFAS are involved in cholesterol metabolism, however, the mechanisms underlying the associations are poorly understood.

**Objective:**

We aimed to evaluate associations of plasma PFAS with detailed lipid and lipoprotein subfractions in an adult population of men and women.

**Methods:**

We measured concentrations of cholesterol and triglycerides in lipoprotein subfractions, apolipoprotein subclasses, as well as fatty acid and different phospholipid measures, using serum proton nuclear magnetic resonance (1H-NMR), and four plasma PFAS using liquid chromatography-mass spectrometry (UHPLC-MS/MS). Measurements were available for 493 participants (all aged 50 years, 50% female). Multivariable linear regression was used to estimate the association of four PFAS with 43 different 1H-NMR measures, with adjustment for body mass index (BMI), smoking, education, and physical activity.

**Results:**

We found that perfluorooctanesulfonic acid (PFOS), perfluorooctanoic acid (PFOA), perfluorodecanoic acid (PFDA), but not perfluorohexanesulfonate (PFHxS), concentrations were consistently positively associated with concentrations of cholesterol in lipoprotein subfractions, apolipoproteins, as well as composite fatty acid- and phospholipid profiles. The most consistent associations were found for the relationship of PFAS with total cholesterol in intermediate-density lipoprotein (IDL), across all low-density lipoprotein (LDL) subfractions and small high-density lipoprotein (HDL). Moreover, we found weak to null evidence for an association of any of the measured 13 triglyceride lipoprotein subfractions with PFAS.

**Conclusions:**

Our results suggest that plasma PFAS concentrations are associated with cholesterol in small HDL, IDL and all LDL subfractions, as well as apolipoproteins and composite fatty acid and phospholipid profiles but to a lesser extent with triglycerides in lipoproteins. Our findings draw attention to the need for more detailed measurements of lipids across various lipoprotein subfractions and subclasses in assessing the role of PFAS in lipid metabolism.

**Impact:**

- By performing an in-depth characterization of circulating cholesterol and triglycerides in lipoprotein subfractions, apolipoprotein, fatty acid, and phospholipid concentrations, this study has expanded upon the limited literature available on the associations of plasma PFAS concentrations beyond clinical routine laboratory testing for lipids.

## Background

Perfluoroalkyl substances (PFAS) constitute a vast group of substances with variable carbon chain length, molecular weight, degree and pattern of fluorination, and polar functional groups [1]. PFAS are chemically resistant with surface tension lowering properties. These characteristics have led to the usage of PFAS in a wide variety of commercial applications such as coatings for clothing, carpets as well as food contact paper; fire-fighting foams; paints, waxes, polishes; industrial surfactants, emulsifiers, and coatings; and personal care products [1]. Due to the extensive field of application, PFAS are released into the environment during production, use and disposal of products [2]. Owing to their environmentally persistent properties, PFAS are found in water, wildlife, and humans as they have relatively high solubility and mobility. Humans are exposed to PFAS through foods [1], drinking water, occupational exposure [3, 4], transfer from mother to children both prenatally (in utero) and postnatally (via breastfeeding) [5], transfer from different packages into foods [6], and dust ingestion and indoor air inhalation [7]. In all cases, transfer rates are dependent on the type of PFAS (chain length, functional group) as well as differences between species and organs.

In humans, PFAS absorb readily in the gastrointestinal tract, distribute in plasma and other organs, and accumulate in the liver [1]. Estimated human half-lives for PFAS are dependent on the chemical functional group, the chain length and interactions with transporters involved in the reabsorption process. Short-chain PFAS have half-lives between a few days to one month, while half-lives for long-chain PFAS can be several years [8]. Due to their exceptional stability in the environment, there has been concern about their potential risks to human health. Epidemiological studies have highlighted positive associations of PFAS with increased circulating total cholesterol and low-density lipoprotein (LDL) cholesterol and lipoproteins [9, 10]. From this perspective, the European Food Safety Authority Panel on Contaminants in the Food Chain (EFSA CONTAM Panel) has emphasized increased serum total and LDL cholesterol as critical outcomes [1]. Although PFAS have been both cross-sectionally and longitudinally associated with total cholesterol and LDL cholesterol in >20 discrete populations [9, 11–20], the associations of PFAS with lipids and lipoproteins currently not included in clinical routine laboratory testing for lipids remain unclear. Thus, the main aim of the current study was to investigate the associations of PFAS concentrations with detailed lipid and lipoprotein subfractions measured by absolute quantification proton nuclear magnetic resonance spectroscopy (1H-NMR).

## Methods

### Study participants

The Prospective study on Obesity, Energy, and Metabolism (POEM) recruited 50-year-old men and women from the general population by a random invitation by mail using public population registers for the municipality of Uppsala, Sweden [21]. The participants received their invitation one month after their 50th birthday. A total of 502 individuals took part in the study, a participation rate of 25%. The study was approved by the ethics committee at Uppsala University (No. 2009/057 and No. 2012/143), and the participants gave their informed consent. All participants were requested to fast from midnight before the investigation that took place in the morning between 8 and 10 am. All participants had to confirm that they adhered to the fasting request before blood sampling, whereafter serum and plasma were separated and stored in −80 °C freezers until analysis.

### PFAS analysis

Target PFASs were determined in 150 µl serum samples using matrix matched isotope dilution ultra-performance liquid chromatography (UPLC-MS/MS, Waters Corporation, Milford, USA) coupled to tandem mass spectrometry as previously described [22]. Native calibration standards and corresponding labeled internal and recovery standards (13 C) were purchased from Wellington Laboratories (Guelph, Ontario, Canada). For PFOS we have analysed and quantified both the linear isomer (L-PFOS) and the total PFOS (including the peak for both branched and linear PFOS) and found that the spearman correlation between these two measures is 0.97. Since all our standards (calibration standards, extraction standard, and injection standard) are L-PFOS we have chosen to present the concentrations for L-PFOS. For quantification, each analytical batch of authenthic samples was processed together with an eight-point matrix matched calibration (seven matrix matched calibrations: R2 > 0.999 and mean RSDs: Perfluorohexanesulfonate (PFHxS) = 13.4%, perfluoro-1-octanesulfonate (L-PFOS) = 11.0%, perfluoro-n-octanoic acid (PFOA) = 7.00%, PFDA = 10.3%). For quality assurance and quality control, 15 NIST SRM 1957 serum samples were processed throughout the study and found to conform with the certified values (mean values and RSDs; PFHxS = 3.70 ngmL^−1^ and 6%; L-PFOS = 11.77 ngmL^−1^ and 7%; PFOA = 4.84 ngmL^−1^ and 4%; PFDA = 0.29 ngmL-1 and 9%). For quality precision, 49 in-house reference plasma samples were processed throughout the study (mean RSDs: PFHxS = 11.9%, L-PFOS = 10.5%, PFOA = 8.60%, PFDA = 14.4%). For method detection limits (MDLs), 49 water blanks were processed throughout the study and the established MDLs were PFHxS = 0.06 ngmL^−1^; L-PFOS = 0.02 ngmL^−1^; PFOA = 0.11 ngmL^−1^; PFDA = 0.10 ngmL^−1^. Overall, the method produced satisfactory results in terms of linear range, accuracy, precision, and sensitivity throughout the study.

### Lipid, lipoprotein, and apolipoprotein analyses

Absolute quantitation plasma lipid, lipoprotein, and apolipoprotein profiling was acquired using an 1H-NMR spectroscopy-based methodology from Nightingale Health. The lipoprotein fractions measured are characterized by particle size; very-low-density lipoprotein (VLDL) fraction consists of extremely large (average diameter >75 nm), very large (64 nm), large (53.6 nm), medium (44.5 nm), small (36.8 nm) and very small (31.3 nm) particles. Intermediate-density lipoprotein (IDL) particles are on average 28.6 nm in diameter. LDL particles are divided into three fractions: large (25.5 nm), medium (23.0 nm) and small (18.7 nm). High-density lipoprotein (HDL) fraction consists of four fractions: very large (14.3 nm), large (12.1 nm), medium (10.9 nm) and small (8.7 nm). The fatty acid profiles included nine fatty acid measures (total, saturated, monounsaturated, polyunsaturated, omega-6 and omega-3 fatty acids as well as degree of unsaturation, linoleic acid, and docosahexaenoic acid). The phospholipid profiles included five composite measures of phospholipids (sphingomyelins, total cholines, phosphatidylcholines, total phosphoglycerides, and triglycerides/phosphoglycerides). The triglyceride fractions measured are characterized by particle size as lipoprotein fractions. The apolipoprotein profile included three composite measures such as Apo B, Apo A1 and Apo B/Apo A1.

### Statistical analysis

PFAS concentrations were not normally distributed while some of the lipid and lipoprotein were, and others were not. Therefore, we first log-transformed the PFAS, lipoproteins, apolipoproteins, and lipid concentrations to achieve normal distributions for all measures. With several exposures evaluated with different distributions (as in the present study with several PFAS), the estimates cannot be easily compared. Therefore, by performing mean centering and unit variance scaling in the data pre-processing stage, variables with different units or distributions can be compared on a level ground. All the variables have the same inherent importance in subsequent linear regression models and the effect estimates are expressed per one SD change. Second, following data pre-processing, a linear regression model was constructed for each metabolic measure, using the lipoproteins, lipids, and apolipoproteins as the dependent variables and the PFAS as the independent variable. We adjusted for sex, body mass index (BMI), alcohol consumption, smoking, education, and physical activity (exercise) with the intention to capture most of the confounding related to a healthy life-style. Last, we evaluated if there were any major sex-differences regarding PFAS vs lipid relationships by including a PFAS*sex multiplicative interaction term in the models described above. All participants were of the same age. All calculations were performed using STATA16 (Stata Inc., College Station, Texas, USA). Statistical significance was defined as false discovery rate *P*_*FDR*_ < 0.05.

## Results

Demographic characteristics of the participants included in the present study are shown in Table 1. In total, 493 participants from Uppsala, Sweden with available PFAS and 1H-NMR metabolomics data were included at the baseline. The mean age was 50 (SD: 0.1) years, 247 (50%) were female, mean BMI was 26.4 (SD: 4.2), and 9.8 % were smokers.

**Table 1:** Basic characteristics of the study participants in the POEM cohort.

*N*	493
Age, years, mean (SD)	50 (0.1)
Female sex (%)	50%
Systolic blood pressure (mmHg), mean (SD)	125.6 (16.4)
Diastolic blood pressure (mmHg), mean (SD)	77.0 (10.1)
HDL-cholesterol (mmol/l), mean (SD)	1.3 (0.3)
Triglycerides (mmol/l), mean (SD)	1.2 (0.9)
BMI (kg/m^2^), mean (SD)	26.4 (4.2)
Waist circumference (cm), mean (SD)	92.5 (11.4)
Fasting glucose (mmol/l), mean (SD)	4.9 (0.9)
Diabetes medication (%)	0.2%
Antihypertensive medication (%)	8.1%
Exercise habits, 4-grade scale, mean (SD)	2.8 (1.01)
Education, years, (%)	<10 years: 8% 10–12 years: 44% >12 years: 48%
Smokers (%)	9.8%

**Fig. 1:**
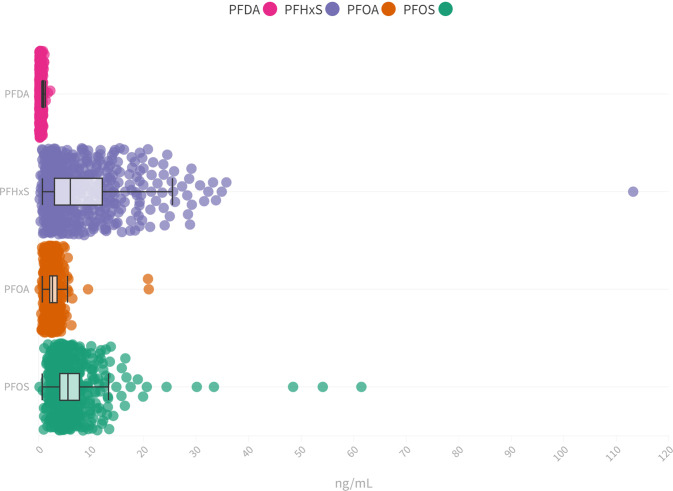
Distribution of investigated PFAS in the study population. Distribution of L-PFOS (median 5.10 ng/mL, IQR 3.50–7.24 ng/mL), PFOA (median 2.14 ng/mL, IQR 1.60–3.0 ng/mL), PFHxS (median 5.5 ng/mL, IQR 2.50–11.61 ng/mL), and PFDA (median 0.34 ng/mL, IQR 0.25–0.45 ng/mL) plasma concentrations in the POEM cohort.

Four PFAS (L-PFOS, PFOA, PFHxS, and PFDA) could be detected in all participants where the highest median concentrations were determined for PFHxS (5.52 ng/mL), followed by L-PFOS (5.07 ng/mL), PFOA (2.13 ng/mL) and PFDA (0.33 ng/mL) (Fig. 1). Although the median concentrations of PFOA and PFDA are similar to those reported in other general populations from Sweden and Europe, the median concentrations of PFHxS and L-PFOS were considerably positively skewed and higher which most likely reflects previous reported findings of an environmental PFAS contamination of drinking water from firefighting foam use on a military base close to Uppsala municipality [23, 24].

**Fig. 2:**
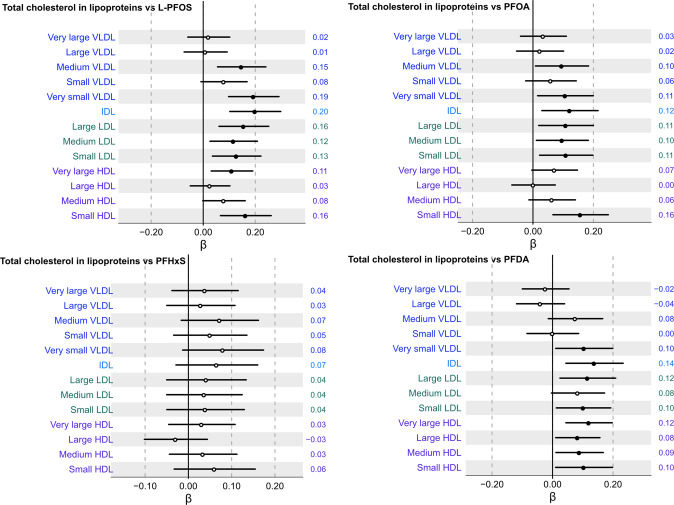
Association between serum concentrations of total cholesterol across thirteen lipoprotein subfractions and L-PFOS, PFOA, PFHxS, and PFDA following multiple adjustment and multiple correction. Beta coefficient intercepts are shown together with 95% confidence interval lines where the black dots indicate statistically significant associations (*P*_FDR_ < 0.05) and white dots non-significant associations, respectively. The estimated beta-coefficients (β) are provided to the right of each row in the forest plot.

We first investigated the association between four major PFAS and thirteen cholesterol lipoproteins subfractions following multiple adjustment (Fig. 2). Among all evaluated subfractions, concentrations of L-PFOS, PFOA and PFDA were significantly positively associated with concentrations of very small VLDL, IDL, small HDL, as well as small, medium, and large LDL subfractions. Across all cholesterol subfractions, positive associations of L-PFOS and PFOA with all measured LDL subfractions were found to be most consistent. PFHxS, however, did not show any significant association with any of the thirteen total cholesterol across lipoprotein subfractions (Fig. 2).

**Fig. 3:**
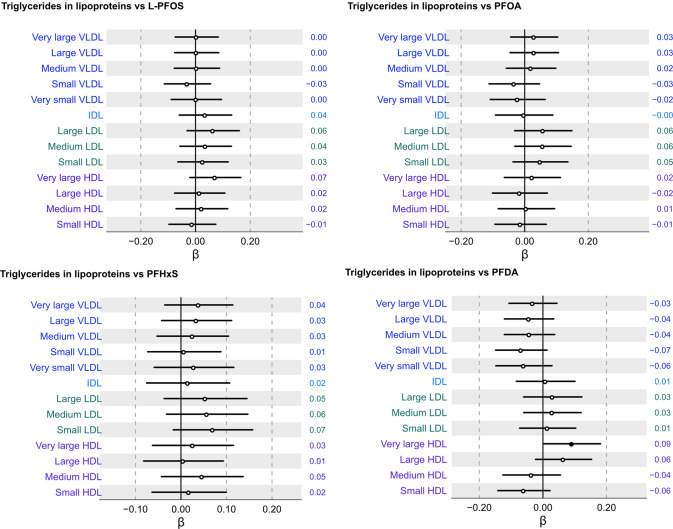
Association between serum concentrations of triglycerides across thirteen lipoprotein subfractions and L-PFOS, PFOA, PFHxS, and PFDA following multiple adjustment and multiple correction. Beta coefficient intercepts are shown together with 95% confidence interval lines where the black dots indicate statistically significant associations (*P*_FDR_ < 0.05) and white dots non-significant associations, respectively. The estimated beta-coefficients (β) are provided to the right of each row in the forest plot.

Next, we evaluated the associations of the four measured PFAS with triglycerides across thirteen lipoprotein subfractions. Figure 3 shows that there were, for the most part, no significant associations with triglycerides across the thirteen different lipoprotein subfractions. Only plasma concentrations of PFDA were found to be positively associated with triglyceride content in the very large HDL fraction. These data indicate that the associations of PFAS and triglycerides across lipoprotein subfractions seem overall weak and largely independent of the multiple positive associations of PFAS with cholesterol across lipoprotein subfractions.

**Fig. 4:**
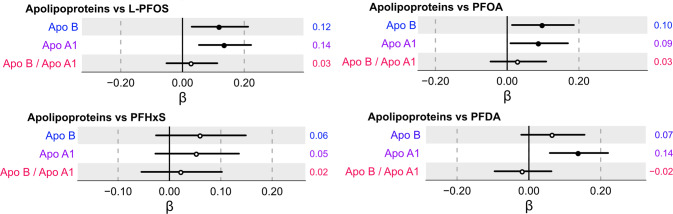
Association between serum concentrations of individual apolipoproteins (Apo B and Apo A1) and the ratio of Apo B/Apo A1 with L-PFOS, PFOA, PFHxS, and PFDA following multiple adjustment and multiple correction. Beta coefficient intercepts are shown together with 95% confidence interval lines where the black dots indicate statistically significant associations (*P*_FDR_ < 0.05) and white dots non-significant associations, respectively. The estimated beta-coefficients (β) are provided to the right of each row in the forest plot.

Subsequently, we investigated the associations of the four PFAS with concentrations of two apolipoproteins Apo B and Apo A1. As illustrated in Fig. 4, higher levels of L-PFOS and PFOA were positively associated with both Apo B and Apo A1 in the multiple-adjusted model, while PFDA was associated with only Apo A1. PFHxS, however, was not associated with the two apolipoprotein measures. Furthermore, no significant associations were observed between the four PFAS and the ratio of Apo B/Apo A1.

**Fig. 5:**
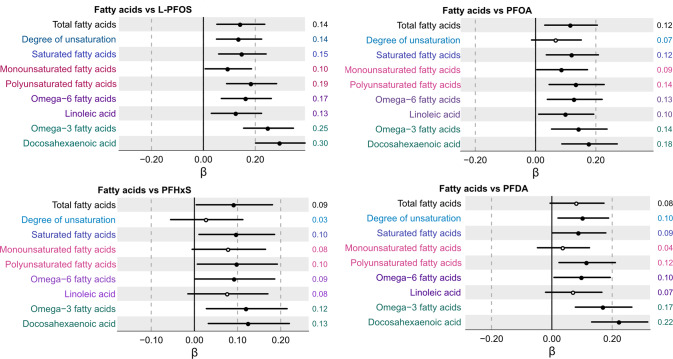
Association between serum concentrations of individual and composite fatty acid measures and L-PFOS, PFOA, PFHxS, and PFDA following multiple adjustment and multiple correction. Beta coefficient intercepts are shown together with 95% confidence interval lines where the black dots indicate statistically significant associations (*P*_FDR_ < 0.05) and white dots non-significant associations, respectively. The estimated beta-coefficients (β) are provided to the right of each row in the forest plot.

Investigation of the associations between plasma PFAS concentrations and nine measures of fatty acid profiles indicated multiple positive associations. As shown in Fig. 5, plasma L-PFOS concentrations were positively associated with all nine fatty acid measures including total fatty acids, degree of unsaturation, saturated fatty acids, monounsaturated fatty acids, polyunsaturated fatty acids, omega-6 fatty acids, linoleic acids, omega-3 fatty acids, and lastly docosahexaenoic acid (DHA). Similar results were also observed for associations with PFOA (Fig. 5). Plasma PFHxS and PFDA concentrations followed a similar trend and were found to be significantly positively associated with six out of nine of the composite fatty acid profiles.

**Fig. 6:**
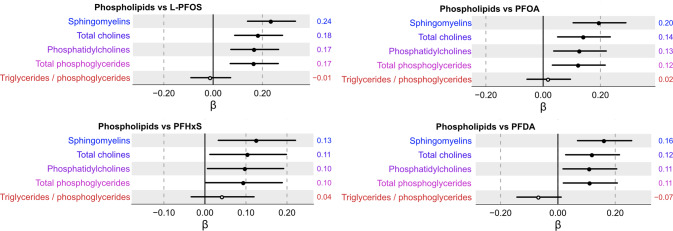
Association between serum concentrations of composite phospholipid measures and L-PFOS, PFOA, PFHxS, and PFDA following multiple adjustment and multiple correction. Beta coefficient intercepts are shown together with 95% confidence interval lines where the black dots indicate statistically significant associations (*P*_FDR_ < 0.05) and white dots non-significant associations, respectively. The estimated beta-coefficients (β) are provided to the right of each row in the forest plot.

An evaluation of the associations between plasma PFAS and five composite phospholipid profiles shows that L-PFOS, PFOA, PFHxS, and PFDA were positively associated with sphingomyelins, total cholines, phosphatidylcholines, and total phosphoglycerides (Fig. 6). No significant association was observed between any of the four PFAS and the ratio of triglycerides/phosphoglycerides.

Lastly, when a PFAS*sex multiplicative interaction term was included in the models described above to test for major sex-differences, no such significant sex-interactions were found following *p* value correction with *P*_*FDR*_ < 0.05.

## Discussion

In this cohort, consisting of middle-aged men and women in Sweden, L-PFOS, PFOA and PFDA concentrations were consistently positively associated with serum concentrations of cholesterol in lipoproteins subfractions, apolipoproteins, as well as composite fatty acid- and phospholipid profiles. Moreover, associations were found to be the most consistent for L-PFOS, PFOA, and PFDA with total cholesterol in small HDL, IDL and small, medium, and large LDL lipoprotein subfractions. Interestingly, none of the evaluated cholesterol in lipoprotein subfractions studies showed significant association with plasma PFHxS concentrations. Triglycerides in lipoprotein subfractions showed null to weak associations with all PFAS. The apolipoproteins (ApoB and ApoA1) and the composite fatty acid and phospholipid profiles were, to a large extent, positively associated with plasma PFAS concentrations. By performing an in-depth characterization of circulating cholesterol and triglycerides in lipoprotein subfractions, apolipoprotein, fatty acid, and phospholipid concentrations, this study has expanded upon the limited literature available on the associations of plasma PFAS concentrations beyond routine clinical lipid markers as reported in previous studies.

Total cholesterol in small, medium, and large lipoprotein subfractions was found to be positively associated with PFAS exposure, with the most consistent association observed for L-PFOS and PFOA. These findings agree with findings from multiple cross-sectional and longitudinal studies concerning associations of clinical routine laboratory lipid testing, where associations of PFAS with LDL-cholesterol or total cholesterol has been consistently observed in >20 discrete populations [9–13, 15–18, 20, 25, 26]. In recent cross sectional and longitudinal studies of associations between PFOS, PFOA and PFHxS and serum lipids in large Swedish adult populations with and without contaminated drinking water, positive associations between PFAS exposure and total cholesterol and LDL were reported [9, 16].

Elevated LDL-cholesterol has been reported to play a central role in atherogenesis and is independently associated with increased risk of cardiovascular disease in observational studies. Further, differences among the LDL-cholesterol containing lipoprotein subfractions have also been reported, showing that small LDL- cholesterol subfractions are associated with a greater cardiovascular disease (CVD) risk than large LDL [27, 28]. This difference is attributed to the higher affinity of small LDL to the LDL receptors, greater binding potential to intra-arterial proteoglycans, and higher susceptibility to oxidation [29–31] as compared to large LDL. Since the introduction of statins 2–3 decades ago and the documented efficacy of those agents [32], the use of statin treatment have increased substantially and reduced the incidence rate of myocardial infarction. Thus, this medical intervention has clearly shown the importance of keeping LDL-levels low in the population.

We also found that PFAS were related to cholesterol content of small HDL, but not consistently to the content in medium, large, and very large HDL except for PFDA that was significantly associated with all four total cholesterol in HDL subfractions. In a study evaluating a detailed lipoprotein analysis and future CVD [33], it was mainly the large HDL particles, not the small HDL particles, that were negatively related to myocardial infarction and stroke. Thus, if the relationships observed in the present study are causal, the somewhat paradoxical increase in HDL associated with PFAS levels may not have any protective impact on future CVD since PFAS increase the levels of HDL particles not being protective for CVD. So far, mixed results have been reported in the literature regarding PFAS levels and CVD in population-based studies [34–43].

We found weak to null evidence for an association between triglycerides in 13 lipoprotein subfractions with the studied PFAS. Similar results have also been reported in other epidemiological studies adults [1, 9, 10]. We found positive associations of PFAS with Apo B and Apo A1. These results in agreement with previous studies of adults and adolescent populations [20, 25, 44]. In a study of National Health and Nutrition Examination Survey for 2007–2014, it was also found that the association of PFAS and Apo B was more pronounced in nondiabetic patients who are not taking lipid-lowering medications [44]. Apolipoproteins play an important role in lipid metabolism by increasing lipid solubility and participating in uptake, assembly, and clearance of lipids. Due to these aspects, apolipoproteins have been considered to have a possible role in the development of CVD, making the association between PFAS and these molecules important in the evaluation of the potential risk of PFAS to human health. Apo B is known to be primarily presented as the protein component of LDL, and Apo A1 as the main protein component of HDL. Since these lipoprotein subfractions have been associated with opposite characteristics in CVD then the association of PFAS with Apo B to Apo A1 ratio (Apo B/ApoA1) would be indicative as a risk factor. Although the current study found positive associations between PFOS and PFOA with both Apo B and Apo A1, none of the examined PFAS was positively associated with the ratio of ApoB/ApoA1.

We observed positive associations of PFAS with composite fatty acid profiles. Several previous studies of adult populations have indicated that PFAS are associated with fatty acid metabolism [45, 46] and the current study provides more insight into the associations of four examined PFAS exposures with saturated and several unsaturated fatty acid subclasses including mono- and polyunsaturated fatty acids, linoleic acid, omega-6 and omega-3 fatty acids as well as DHA. As all these fatty acids present different functions in the organism based on their structure, chain length and configuration, results from the current study suggest that the association of PFAS with different fatty acids as particularly important in the understanding the potential risk of PFAS to human health. Most examined fatty acids are known to be essential as they cannot be synthesized de novo due to the lack of necessary enzymes and therefore, must be derived through diet. Some of these fatty acids are derived from oily fish products that are also known to accumulate PFAS [47, 48]. It could explain the significant positive associations observed in the current study. Furthermore, the present study examined the association between phospholipid subfractions and PFAS exposure, indicating positive associations of sphingomyelins, total cholines, phosphatidylcholines and total phosphoglycerides with all four examined PFAS – L-PFOS, PFOA, PFHxS and PFDA. These findings agree with previous human as well as experimental studies that propose that the penetration of PFASs into the phospholipid bilayer of cell membranes and the disturbance and detachment of phospholipids [49–51] may be one mechanism by which PFAS are involved in lipid metabolism.

We also assessed sex differences in the association between PFAS and lipid and lipoprotein profiles. Since the sample is not very large, we would have run into severe power problems when splitting the sample in two halves given that we needed to adjust for 4*34 PFAS vs lipid and lipoprotein tests. Therefore, as an alternative approach, we included a PFAS*sex multiplicative interaction term in the models to test for major sex-differences, but no such interaction was found to be significant. It would however be warranted to perform a sex-stratified analysis in future studies with larger sample size, since circulating levels of PFAS could be different in men and women [52].

The strength of this study is the extensive characterization of human lipid metabolism using a state of the art 1H-NMR platform in fasted blood samples, including lipid measures not reflected by routine clinical lipid parameters which make this study unique in comparison to previous studies. The limitations of this study include small population size, the cross-sectional study design, as well as the inability to account for potential confounders such as food intake (dietary habits) and socio-economic position. Lastly, POEM is an adult ethnically homogeneous cohort, limiting the ability to generalize results across other populations and age groups. Therefore, more research is needed to assess the generalizability of our findings to new data, for example, including a more diverse range of ages and ethnicities.

## Conclusion

This study investigated the relationship between plasma PFAS concentrations and a detailed characterization of human lipid metabolism including concentrations of cholesterol and triglycerides across lipoprotein subfractions, apolipoprotein subclasses, as well as fatty acid and phospholipid measures. We observed that L-PFOS, PFOA and PFDA concentrations were consistently positively associated with serum concentrations of cholesterol in lipoproteins subfractions, apolipoproteins, as well as composite fatty acid- and phospholipid profiles. Moreover, associations were found to be the most consistent for L-PFOS, PFOA, and PFDA with total cholesterol in small HDL, IDL and small, medium, and large LDL lipoprotein subfractions. Interestingly, none of the evaluated cholesterol in lipoprotein subfractions studies showed significant association with plasma PFHxS concentrations. Apolipoproteins (ApoB and ApoA1) and the composite fatty acid and phospholipid profiles to a large extent were positively associated with all four plasma PFAS concentrations. Lastly, triglycerides in lipoprotein subfractions showed null to weak associations with all four evaluated PFAS. In conclusion, our findings draw attention to the need for more detailed measurements of lipids across lipoprotein subfractions in assessing the role of PFAS in lipid metabolism.

## Data Availability

The datasets generated and/or analysed in the current study are not publicly available but are available from the corresponding author upon reasonable request.
